# Supplementary material for: Analysis of PPARγ Signaling Activity in Psoriasis
Source: Int J Mol Sci. 2021 Aug 10;22(16):8603. doi: 10.3390/ijms22168603 (PMC8395241; doi:10.3390/ijms22168603)
Supplement: Supplementary file 1 [file ijms-22-08603-s001.zip › Supplemental materials_Analysis of PPARg signaling activity in psoriasis/Pathway models/Models images and html files/PPARG inhibits phenotypes assotiated with psoriasis/125184.html]

hyperglycemia


# Disease hyperglycemia

|  |  |
| --- | --- |
| URN | urn:agi-meshdis:Hyperglycemia |
| Total Entities | 1 |
| Connectivity | 7119 |
| Name | hyperglycemia |

---

|  |  |
| --- | --- |
| ChildConcepts | postprandial hyperglycemia |

---

|  |  |
| --- | --- |
| Pathway | PPARG inhibits phenotypes assotiated with psoriasis |
|  | efects selected |

---

|  |  |
| --- | --- |
| MedScan ID | 9010776 |

---

|  |  |
| --- | --- |
| Alias | hyperglycemic syndromes |
|  | Blood Glucose, High |
|  | Hyperglycemia |
|  | hyperglycemic syndrome |
|  | Glucose, High Blood |
|  | hyperglycemic |
|  | hyper-glycemic |
|  | Hyperglycemias |
|  | hyperglucemia |
|  | High Blood Glucose |
|  | hyper-glycemia |

---

|  |  |
| --- | --- |
| MeSH ID | D006943 |
|  | M0010824 |

---

|  |  |
| --- | --- |
| MeSH Heading | Hyperglycemia |

---
